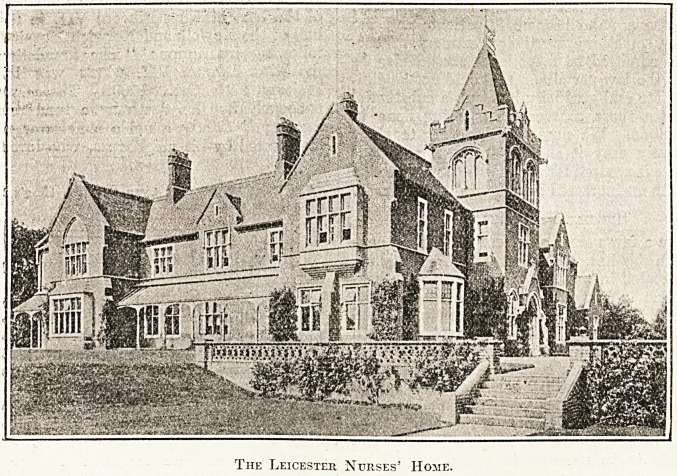# Reports on Hospitals of the United Kingdom

**Published:** 1914-01-03

**Authors:** Henry Burdett


REPORTS ON

### Hospitals of the United Kingdom.

By SIR HENRY BURDETT, K.C.B., K.C.V.O.

SERIES III.

### THE NORFOLK AND NORWICH HOSPITAL, NORWICH.

FOUNDED in 1771, the rebuilding of this hospital was undertaken in 1879 and completed in 1884. This spacious, well-planned, and attractive hospital contains 230 beds, of which 202 are always available, seventeen beds are kept for emergency use, eight beds are set apart for infectious cases, two for nurses, and one as a twelve-hour room. These beds are worked by a staff of forty-four staff nurses and probationers. The wards have central fireplaces of an old but fairly effective pattern, though the grates might be improved and modernised by substituting slow-combustion shell-shaped grates for the straight bars at present in use. This alteration would probably cost about £6 per grate, but the outlay should be speedily repaid by an economy in the consumption of coal, and an increase in the heating and brightness of the wards. The ward space is ample; the wards are well-lighted, but a saving might have been effected in the original cost and in the efficiency of the ventilation had their height been reduced to 12 feet, instead of being 2 feet higher.

NORFOLK AND NORWICH HOSPITAL—FRONT ELEVATION.

### MINOR POINTS, GOOD AND OTHERWISE.

The type of locker is not good, and the Cromer pattern might be substituted with advantage and at relatively small cost. We think, too, that a good ward table, containing drawers and cupboards for the reception of dressings and other necessary articles in constant use, would help the easy administration of the larger wards, and be found an advantage in many ways. We would also suggest that each bed should have a chair beside it for the use of the patient's friends and for the patient's own use when able to get up. These additions should afford an opportunity to some thoughtful and benevolent people who are interested in the hospital who may like, by gifts of articles of this kind, to identify themselves, or some members of their families, with its work. The original contractors have done less than justice to their reputation and to that of the architect by the quality of much of the wood used for the floors, especially in the corridors. In due course, as opportunity offers, it would be desirable to substitute American willow or teak throughout for the floors of the wards and corridors. In the bathrooms metal cupboards have been placed, and through them is passed a steampipe with the object of keeping the bedpans, which are stored there, ready for use and in a condition to increase the comfort of the patients. The idea is a good one, but to make it effective small radiators should be added in the form of metal tubes, which might take the place of the solid metal racks they at present contain.

### KING EDWARD VII. WARDS.

A striking feature of the present hospital is the King Edward VII. wards. The design, planning, construction, and detailed work connected with these wards are highly creditable to all concerned. They are deservedly regarded with pride by the administration. On the ground floor are wards for septic cases, with ample verandah space so that patients' beds can be wheeled out and the patients treated under very favourable conditions. The results are stated to have been most satisfactory, and it is evident that those responsible have spared neither thought, nor experience, nor technical knowledge, in making them as complete and perfect as possible. On the upper floor are placed isolation cubicles separated by glass partitions carried to the top of the ward ceilings, each of them being self-contained and the whole open to the observation of the staff on duty. We found them very complete and they include accommodation for the nursing staff employed, so that the whole of this section of the hospital is self-contained and up to date. The opening of wards for septic cases—which have their own operation theatre in connection with them, and contain an isolation ward for the reception of any special cases so that it may be used as a precaution and safeguard whenever necessary—has not only provided sufficient accommodation for the reception of all septic cases from the general wards, but has enabled many additional patients to be received into the hospital, who would otherwise have had to be retained on the waiting list.

### The Operation Theatre.

As originally planned, the hospital, when first opened, had one very large operation theatre which, however, did not fulfil the necessary requirements. The medical staff, largely, we understand, under the guidance and suggestion of Mr. Ballance, one of the surgeons, proposed, and the committee sanctioned, the reconstruction of this portion of the hospital. Two new theatres were planned and built behind the old theatre, which was broken up into sections—one section being used as an anæsthetic room. Between the new theatres is placed a large, airy room where all the washing-up and other necessary work can be carried on, so as to keep each theatre aseptic and as hygienically perfect as possible. This scheme, which has recently been completed at considerable cost, abundantly justifies the money spent upon it, and has greatly facilitated the work of this department, its smooth administration and efficiency. The new theatres could not be provided with the usual north light, but, although they may occasionally become heated in the warmer portions of the year when the sun is at its full height, any trouble of this kind is provided against by outside blinds, "summer cloud," and electric fans acting as extractors. The medical staff and the governors are to be congratulated upon the boldness of the scheme and its success.

### The Domestic and Nursing Departments.

The nurses are accommodated outside in a home specially built for the purpose, which contains some excellent reading and recreation rooms and a number of single rooms for the nursing staff. The nurses' recreation room contains something like an adequate number of comfortable seats and chairs, a feature not always found in nurses' homes of a type so recent, and we have the hope that wherever in other hospitals these defects exist they will speedily be remedied. The long hours, under the most favourable conditions, that nurses have to remain on duty, and the arduous nature of much of their work, entitle them to have ample provision made for their comfort in off-duty hours. We congratulate the Norwich authorities upon the example they have set by the sound provision they have made for their nurses. We congratulate them too on the larger dining-room for the nursing staff, which forms one of the most recent and admirable additions to the establishment. We have not space to-day to deal fully with the excellent system of nursing Miss Cann has so cleverly organised. Cleverly, because her special difficulties, as we may show in a subsequent article, were not few. It must suffice to offer Miss Cann sincere congratulations on her zeal and success, whilst we commend for general adoption her plan of keeping a Special Rest Room at the Convalescent Home at Cromer exclusively for the members of the nursing staff.

### The General Administration.

There is a counterpart to the Norfolk and Norwich Hospital, so far as the plan is concerned, in another county town which we will not mention here because the contrast between the two institutions as we have seen them was so striking. In the twin hospital the administration was imperfect, and the general condition throughout suffered materially in consequence. Indeed, we began to think that the plan was too extended for a county hospital, and that the cost of maintenance was so increased as to render it relatively impossible for a county hospital committee to defray the cost of keeping everything smart and up to date. We therefore looked forward with special interest to the inspection of the Norfolk and Norwich Hospital, which we made on October 22, 1913. A careful inspection proved to demonstration that it was not cost of upkeep but feebleness of administration which might make a hospital plan like that at Norwich so disappointing to the administrator. The county and city of Norwich have the immense advantage of possessing officials who are animated by the spirit of personal service for the sick to a very unusual extent, and to that fact is no doubt due the splendid efficiency of the administration, and the keen alertness of everybody on the staff, both male and female, upstairs and downstairs, whom it was our privilege to meet. We wished to see the new dining-hall, and as lunch was proceeding there we gladly accepted the matron's invitation to join the sisters' table and sample the viands. It was a well-served, simple, but enjoyable meal, both the food and company contributing much to our pleasure as a guest. On a similar occasion in an American hospital everything served at the meal was prepared and cooked by the nurses. All are properly proud of their hospital at Norwich, interested in their work, and wide-minded enough to take an interest in the work of others up and down the country. The wards are attractive, the control exercised over important matters affecting the discipline, the ventilation, the upkeep, and the comfort of the patients is good, and we congratulate the governors, the subscribers, and the people who have the privilege of being served by this great county hospital, which nobly ministers to the necessities of the greater portion of Norfolk, in the heart of which it is situated. We were particularly pleased with the smart and orderly arrangement of the stores, the quality of the food, and the general control exercised over these important details of hospital management.

### A Notable Report.

The report of this hospital must give pleasure to every one in proportion to his knowledge of such publications and the things which matter as an indication of the quality of the work and the control and interest exercised by the committee and the secretariat. It can truly be said of this report, it is so arranged and so full of information that an expert after perusing its pages could describe in an interesting way the progress, development, changes, relative efficiency or otherwise, and the many special departments of the institution from the date of its foundation in 1808 to the present time. The only thing which surprises us about it is that a relatively new departure, which reflects honour upon the women of the county of Norfolk—*i.e.*, the Ladies' County Associations, which raised £380 during 1912—is not given a prominent position by having a separate line on the income side of the audited accounts. Is this because the accounts are kept on the uniform system and Norwich is the first county hospital to possess the honour of having the invaluable help of several Ladies' Associations? We spent a quarter of an hour without success in endeavouring to find out under what total the women's contribution of £380 had been credited in the accounts. In the report for 1913 we shall look with interest for a full recognition of the beginning of a movement which, if it spreads, as we hope it will spread, to every village and town throughout Norfolk, may prove one of the most fruitful sources of income.

In this connection we may properly point out the good work which the secretary-superintendent, Mr. Frank G. Hazell, has done and is doing with such commendable success.

### The Financial Position.

We read recently in a newspaper an account of the finances of this hospital, which seemed to imply that it was being starved and kept back by want of funds. Having studied the figures for five years and gone carefully into them, we have formed the conclusion that, if the hospital had an additional income of £2,000 a year it would be able to pay its way. It might even have in hand a little money towards the ever-increasing cost of treatment in the present day. Taking the five years ending 1912 we find that the average deficit in ordinary income has been £2,800. Against this we may fairly set an average annual yield from legacies during the same period of £4,000. Another encouraging feature is that the committee have been able with fair regularity year by year to invest considerable sums. The hospital has at present invested, but free in case of necessity for devotion to general purposes, a sum approaching £24,000, in addition to perpetual and other investments for special purposes, amounting to approximately £100,000. These facts prove the careful and thrifty policy which has characterised the management of this hospital, and should certainly produce the £2,000 a year of extra income at present needed before the end of 1914. This sum must surely be forthcoming when it is pointed out that, under the item of workmen's collections, apart from Hospital Saturday, the entry on the income side is *nil*.

The Leicester Nurses' Home.

WHAT MIGHT READILY BE ACCOMPLISHED.

There are at present, we gather, seven District Ladies' Associations. If Norfolk were divided throughout into districts, there should, we imagine, be at least twenty. The population of Norwich, apart from the county, is upwards of 120,000, but the total sum received from the Hospital Sunday and Saturday Funds combined was only £1,085 in 1912. We are of opinion, judging by the yield of communities of equal size in other parts of the country, that if the Hospital Saturday Fund were placed entirely in the hands and under the administration of the working-men, and if the Hospital Sunday Fund were kept distinct from it and worked by a council of clerical and lay members in equal proportions, the yield from these two sources should readily be increased to at least double the present total. There is, further, a source of revenue which appears to be untouched—namely, insured persons, who become in-patients and are able to pay something for their treatment. Poor-Law cases admitted as in-patients should also be paid for as the law permits. It should not be difficult to initiate and gradually build up a substantial revenue from these sources, as is being done in other hospitals. We urge this in order to show how relatively easy it should prove, if sufficient driving force is secured for the effort, to provide this hospital with an increased income of £2,000 a year.

SPECIAL FEATURES.

There are a few points of special interest in connection with this hospital. First of all, ophthalmic cases are treated in a building adjacent to the hospital site, known as the Grove House, which is the property of the hospital and is let to the Norfolk and Norwich Eye Infirmary at a nominal rent, in return for which service the infirmary undertakes to treat all the ophthalmic cases sent to it by the hospital. By this arrangement the latter is saved the annual cost of maintaining a special eye department. Another pleasant feature to note is that the average attendance of the Board of Management—a fact which ought to be recorded in every hospital report—is remarkably steady and high—*i.e.*, eighteen members are present, at every meeting, on an average. We note that the yield of income to the hospital from the private nursing staff exceeded £2,000 in 1912. We hope that a good proportion of their earnings will soon be devoted, as at Guy's Hospital, to the provision of an adequate annual allowance to each nurse on her retirement, after a fixed number of years' service.

A WELL-ORGANISED ALMONER'S DEPARTMENT.

Pages 19 to 21 of the report are most interesting. They refer especially to the out-patient department, and include the almoner's report. We are glad to note that this hospital has a standard of suitability for hospital treatment which every voluntary hospital ought to have and publish in its report. Suitable cases are, (1) patients sent for special advice and treatment, and (2) patients who are, and always have been, ineligible for a club and cannot afford private attendance, but who are able to maintain themselves or to be maintained, without resort to the Poor Law. Except for special advice and treatment, patients who have made satisfactory provision for medical attendance, or who depend entirely for their maintenance on Poor-Law relief, or can afford to pay for the treatment, or who are unlikely to receive considerable benefit from it, are defined as unsuitable cases for admission. It is an interesting fact, as we gather from the almoner's balance sheet, that the average of cost at Norwich of a set of artificial teeth to the patient or the governors is £4.

THE LATE KING EDWARD VII.

The voluntary hospitals of Great Britain owe more probably to King Edward VII. than to any monarch who has preceded him on the throne. Norfolk is the county in which his beloved home was situated, and it is a pleasure to note that the simple pedestal of the bust of King Edward VII. in the entrance hall of the Norfolk and Norwich Hospital bears the following inscription: "The foundation-stone of the King Edward VII. wards was laid by his Majesty 25th Oct., 1909. These wards were endowed after his death by a fund raised to his memory." Truly a noble benefactor nobly commemorated by his neighbours, who loved him.

**The West Norfolk and Lynn Hospital, King's Lynn, will be reported on next week.**

## Figures and Tables

**Figure f1:**
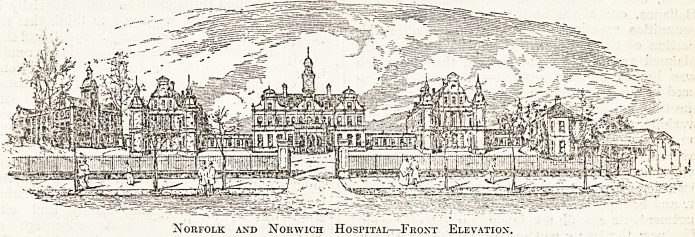


**Figure f2:**